# Managing severe infection in infancy in resource poor settings

**DOI:** 10.1016/j.earlhumdev.2012.09.005

**Published:** 2012-12

**Authors:** Anna C. Seale, James A. Berkley

**Affiliations:** KEMRI-Wellcome Centre for Geographic Medicine and Research — Coast, PO BOX 230, Kilifi 80108, Kenya; Centre for Clinical Vaccinology and Tropical Medicine, Oxford University, Churchill Hospital, Oxford OX3 7LE, UK

**Keywords:** Infant, Neonatal, Newborn, Infection, Management, Resource-poor

## Abstract

Reducing childhood mortality in resource-poor regions depends on effective interventions to decrease neonatal mortality from severe infection, which contributes up to a half of all neonatal deaths. There are key differences in resource-poor, compared to resource-rich, countries in terms of diagnosis, supportive care and treatment. In resource-poor settings, diagnosis is based on identifying clinical syndromes from international guidelines; microbiological investigations are restricted to a few research facilities. Low levels of staffing and equipment limit the provision of basic supportive care, and most facilities cannot provide respiratory support. Empiric antibiotic treatment guidelines are based on few aetiological and antimicrobial susceptibility data. Research on improving health care systems to provide effective supportive care, and implementation of simple pragmatic interventions, such as low-cost respiratory support, are essential, together with improved surveillance to monitor emerging drug resistance and treatment failures. Reductions in mortality will also be achieved through prevention of infection; including emerging vaccination and anti-sepsis strategies.

## 1 Introduction

Whilst substantial progress has been made in reducing childhood mortality in the under 5s in resource-poor regions, progress in reducing deaths in the neonatal period (< 28 days), has been limited. Globally, under-5 mortality has declined by 28%, from 90 deaths per 1000 live births in 1990, to 65 in 2008 [1]. Reductions in neonatal mortality have, however, been considerably less, and are slowest in regions with the highest burden, South-east Asia and Africa; Africa's neonatal mortality only decreased 17.6% (43.6 to 35.9 per 1000 live births) between 1990 and 2009 [2]. Thus there is an epidemiological transition in childhood mortality, with neonates representing an increasing proportion (41%) of the global burden of childhood deaths [3]. In order to make further progress towards Millennium Development Goal 4 of reducing childhood mortality by two thirds between 1990 and 2015, neonatal mortality must be reduced.

Here we aim to provide an overview of some of the key differences in management of severe infection in resource-poor compared to resource-rich settings, and consider research directions to improve management and to prevent infection. We restrict this paper to severe bacterial infection since there are limited data on congenital infections such as cytomegalovirus and rubella in resource-poor settings, but these are important areas for future research.

## 2 Causes of neonatal mortality

The principle causes of neonatal mortality are infection, birth asphyxia and preterm birth. In resource-poor settings, infection likely contributes to up to a half of all neonatal deaths [4]. This is due to a high burden of infectious disease, but also high levels of co-morbidity including intrauterine growth restriction (IUGR) and preterm birth: 85% of all preterm births occur in Africa and Asia [5]. In one study in rural India, combinations of preterm birth and sepsis, and IUGR and sepsis, resulted in 35% and 22.5% of neonatal deaths respectively [6]. Maternal HIV infection is also associated with neonatal mortality in resource-poor settings [7]; in both HIV exposed uninfected (HEU) and HIV exposed infected (HEI) infants [8]. This may be in part due to lower specific antibody responses, associated with ante-natal HIV exposure [9]. However, recent data suggest that it is HEI neonates that are most at risk. Secondary analysis of an anti-sepsis trial in South Africa [10] reported much higher rates in HEI than HEU infants of both neonatal early onset sepsis (EOS) (134 vs 21.5 per 1000; P < 0.0001) and late onset sepsis (LOS) (26.8 vs 5.6 per 1000; P = 0.042) [11].

## 3 Diagnosis

**Fig. 1:**
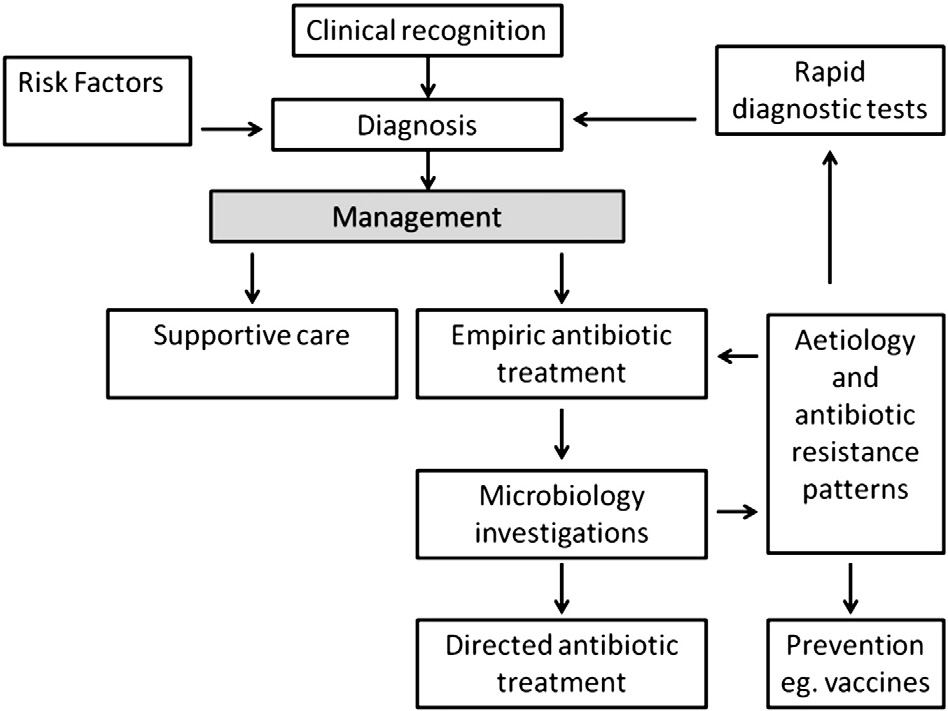
Contributors to optimising management of severe infection in neonates in resource poor-settings.

In resource-poor settings, most sick newborns are not assessed and managed by specialist paediatricians. Clinicians are usually dependent on recognition of patterns of clinical signs and symptoms for diagnosis, which is central to management (illustrated in Fig. 1). Algorithms such as those given in the World Health Organisation (WHO) Integrated Management of Childhood Illness (IMCI) support this approach, together with courses providing training in Emergency Triage Assessment and Treatment (ETAT). These algorithms are a pragmatic approach to detect severe illness with an appropriate index of clinical suspicion; features normally obtained in a history in resource-rich settings, such as maternal pyrexia, prolonged rupture of membranes, and gestation may be unknown. Basic laboratory investigations to support diagnosis, such as C-reactive protein and a full blood count may be unavailable. Most hospitals lack microbiological facilities to detect invasive infection (primarily blood or cerebro-spinal fluid cultures) and high quality microbiological investigations are almost always restricted to a few research settings [12].

Identifying the best clinical predictors of severe disease in young infants in resource-poor settings is therefore essential to developing effective guidelines. In the first WHO multinational study of severe bacterial infection in young infants conducted in Ethiopia, The Gambia, The Philippines and Papua New Guinea [13], fourteen clinical signs and symptoms were identified as key predictors of severe disease [14]. However, the study was limited in terms of identifying early neonatal infection, by the number of neonates in the first week of life included in the study. A more recent, larger WHO multinational study in Bangladesh, Bolivia, Ghana, India, Pakistan, and South Africa recruited 3177 neonates aged 0–6 days and 5712 infants aged 7–59 days. It identified seven factors in neonates aged 0–6 days (severe jaundice excluded), of which the presence of one predicted severe illness, with high sensitivity (85%) and specificity (75%). These were: history of difficulty feeding, history of convulsions, movement only when stimulated, respiratory rate ≥ 60 breaths per minute, severe chest indrawing, and temperature > 37.5 °C or < 35.5 °C. These indicators had similar sensitivity (74%) and specificity (79%) in 7–59 day old infants [15].

There are limitations to the use of clinical signs and symptoms for diagnosis of severe infection. Absence of microbiological culture investigation limits individual management; physicians are unable to rationalise and direct antibiotic treatment, and confirm their clinical diagnoses and there are particular difficulties in confirming the diagnosis of neonatal meningitis, for which signs and symptoms are non-specific. Lumbar puncture is an important diagnostic tool for neonatal meningitis, and can provide useful information even without the availability of microbiological culture [16]. New diagnostic methods which are low complexity and not reliant on conventional culture could make microbiological diagnosis more affordable and feasible in resource-poor settings. They are easily performed, not requiring highly trained, qualified laboratory staff. Examples include rapid immunochromatographic tests, such as those for *Streptococcus pyogenes* (Group A Streptococcus) [17], as well as HIV and malaria. Rapid tests using PCR based methods are also in development.

## 4 Supportive care

Resource-poor settings are limited in their ability to provide basic supportive care (including routine observation, monitoring and documentation) by inadequate staffing (for example 2 trained staff to 100 children is not uncommon), as well as availability of training and equipment. Routine newborn documentation can be inadequate for care. Although simple admission pro forma, such as newborn admission records, can help improve clinical assessment and record keeping [18] improving overall supportive care is challenging and requires strengthening of health systems. A recent cluster randomised trial in eight rural Kenyan hospitals reported that changing practice is best achieved through comprehensive intervention, including evidence-based guidelines, training, job aides, local facilitation, supervision, and face-to-face feedback; when compared to a smaller intervention package of guidelines, didactic training, job aides, and written feedback. Completion of admission assessment tasks was higher in the comprehensive intervention sites at 18 months compared to partial (mean = 0.94 versus 0.65) [19].

Where neonatal care is limited, alternatives to conventional care may be appropriate and beneficial. Kangaroo mother care has been shown to reduce neonatal infection at 40–41 weeks gestational age (typical RR 0.42, 95% CI 0.24 to 0.73 from meta-analysis) in stabilised low birth weight neonates, but more work is needed to establish its use in neonates requiring more conventional neonatal support [20].

A specific and important element of neonatal supportive care, lacking in resource-poor settings, is respiratory support. This is due to deficiencies in equipment, and a lack of training for health care staff. There are, however, methods of providing mild to moderate respiratory support through continuous positive airway pressure (CPAP) which are potentially more feasible and low-cost for use in resource-poor settings. In some cases this can provide an alternative to conventional ventilation [21]. Bubble-CPAP is a variation of conventional CPAP in which gas flows through the expiratory CPAP tubing, submerged underwater to a depth equal to the required positive end expiratory pressure (PEEP) in centimetres. This is an intervention that appears to have great potential, but it is important that bubble-CPAP is used safely in resource poor settings [22], with appropriate training and monitoring, as recently demonstrated in studies in Malawi and Fiji [23,24].

## 5 Antimicrobial treatment

Developing appropriate treatment guidelines and evaluating their efficacy is challenging in resource-poor settings. In particular this relates to the lack of microbiological investigation, which means data on aetiology of neonatal infection and antibiotic sensitivities are scarce. International guidelines from the World Health Organisation, (WHO) Pocketbook of hospital care for Children, in common with guidelines in resource-rich settings, recommend broad-spectrum parenteral antibiotics, usually a combination of penicillin and gentamicin, or a third generation cephalosporin, based on covering common neonatal pathogens.

There are important differences in aetiology of neonatal infection in resource-poor and resource-rich settings however. In resource-rich settings, the leading causes of early onset neonatal sepsis (EOS (0–48 h)) are *Escherichia coli* and *Streptococcus agalactiae* (Group B Streptococcus, GBS). GBS has only recently been identified as a pathogen in EOS in resource-poor settings [25]. *Listeria monocytogenes* is very rarely identified in EOS in resource-poor settings [26], probably due to lower intake of dairy foods than in resource-rich settings. Gram negative infections (in addition to *E. coli*) such as *Klebsiella pneumoniae*, are more common in resource-poor settings. In late onset sepsis (LOS (48 h–28 days)) a key difference is the high incidence of coagulase negative Staphylococci (CONS) in resource-rich settings. This usually occurs in preterm neonates, associated with indwelling devices, such as percutaneous long lines. In resource-poor settings it is unclear whether it is a contaminant, or a pathogen, and the indwelling devices it is associated with are much less commonly used. There are similarities in aetiology too, however, as in both settings LOS is commonly caused by gram negative bacteria, *Staphylococcus aureus* and *Streptococcus pneumoniae* [26,27].

Assessment of continued efficacy of treatment guidelines is essential. In common with resource-rich settings, neonates in resource-poor settings are at increasingly high risk of hospital acquired infection and drug-resistant pathogens [28,29]. Microbiological surveillance of pathogens and their antibiotic susceptibilities in resource-poor settings should be a priority, as well as monitoring of treatment failures using current guidelines.

Improving neonatal care in the community is also important because of difficulties accessing health services in some areas. A large cluster randomised trial (39 intervention and 47 control villages) of a package of home-based neonatal care in rural India, including community treatment of neonatal sepsis, decreased neonatal sepsis case fatality from 16.2 to 2.8% [30].

## 6 Research directions

Research to improve diagnostic methods, pragmatic interventions for supportive care and surveillance of drug-resistance for treatment of neonatal infection in resource-poor settings are high priorities, as described. However, research to prevent severe infection is also important, as discussed below.

Design and implementation of immunisation is a key element of prevention and could build on and strengthen existing maternal immunisation programmes (such as maternal tetanus) in resource-poor settings. Potential vaccine candidates for neonatal sepsis include Group B Streptococcus (GBS), for which a trivalent conjugate vaccine is in phase II clinical trials in pregnant women in South Africa [31]. Data on invasive serotypes of GBS in resource-poor settings are very limited, and more data are needed to inform vaccine development. Current evidence suggests a five-valent vaccine (serotypes Ia, Ib, II, III, V) would cover most GBS disease [25]. Future directions include new research techniques using whole genome sequencing, which are increasing the likelihood of successful identification of new protein targets for vaccines, for both GBS and other important neonatal pathogens such as *E coli* [32].

Antisepsis is another strategy, however vaginal chlorhexidine was shown to be ineffective before delivery in reducing neonatal sepsis in a randomised controlled trial of 8011 women in South Africa [10]. In contrast, anti-sepsis after delivery, at potential portals of infection, such as the umbilical cord, represents a potentially highly effective method of reducing neonatal infection. A factorial, cluster-randomised trial in rural Pakistan, used 4% chlorhexidine solution applied to the cord from birth to 14 days. There were reductions in omphalitis (risk ratio 0.58, 95% CI 0.41–0.82) and neonatal mortality (risk ratio 0.62, 95% CI 0.45–0.85) in those with the chlorhexidine intervention, although hand-washing (another arm of the trial) did not show any effect [33]. In addition, prevention of infection through protection of sites of infection entry has also been demonstrated using skin emollients in preterm neonates. Application of sunflower seed oil or Aquaphor ointment in a randomised control trial of 497 neonates in Bangladesh, resulted in 26% and 32% reductions in neonatal mortality rates compared to no emollient therapy [34].

## 7 Conclusions

Improving management of severe infection in infancy is an international priority. Key factors in improving care in resource-poor settings are improved aetiological diagnosis and effective provision of supportive care, including non-invasive respiratory support. Effective prevention strategies through new vaccines and anti-sepsis interventions are important research directions.

## Conflict of interest

None.
